# A randomized cross-over study of the acute effects of running 5 km on glucose, insulin, metabolic rate, cortisol and Troponin T

**DOI:** 10.1371/journal.pone.0179401

**Published:** 2017-06-16

**Authors:** Boris Keselman, Marta Vergara, Sofia Nyberg, Fredrik H. Nystrom

**Affiliations:** Department of Medical and Health Sciences, Faculty of Medicine and Health Sciences, Linköping University, Linköping, Sweden; Medical Clinic, University Hospital Tuebingen, GERMANY

## Abstract

**Background:**

We aimed to study the impact by running 5 km, at maximal speed, on the normal variations of metabolic variables related to glucose, insulin, insulin sensitivity, cortisol, glucagon, Troponin T and metabolic rate.

**Material and methods:**

Five women and 12 men 25.7±5.2 years of age with a body-mass-index of 22.5±2.3 kg/m^2^ where recruited to run 5 km at individual maximal speed in the morning, and to a corresponding day of rest, followed by standardized breakfast and lunch meals. Blood sampling and measurement of indirect calorimetry were done before and after meals. The participants were randomized regarding the order of the two trial-days in this cross-over study.

**Results:**

Insulin and cortisol levels were higher, and insulin sensitivity was lower, on the race-day compared with the day of rest (linear mixed model: p<0.0001 for all three analyses). However, glucose levels and metabolic rate did not differ between the two trial days (p = 0.29 and p = 0.53, respectively). When analyzing specific time-points we found that glucose increased from 5.01±0.37 mmol/l to 6.36 ± 1.3 mmol/l, p<0.0001, by running, while serum insulin concomitantly increased from 42±21 to 90±54 pmol/l, p<0.0001. In accordance, the QUICKI index of serum sensitivity, 1/(log_10_insulin+log_10_glucose), was lowered post-race, p<0.0001. Serum cortisol levels increased from 408±137 nmol/l to 644±171 nmol/l, p<0.0001, post-race while serum glucagon levels were unaffected. Troponin T was detectable in serum post-race in 12 out of the 17 participants and reached or surpassed the clinical reference level of 15 ng/l in three subjects. Post-race electrocardiograms displayed no pathologies.

**Conclusions:**

Relatively short running-races can apparently induce a reduction in insulin sensitivity that is not fully compensated by concomitantly increased insulin secretion intended to ensure euglycemia. Since also Troponin T was detected in plasma in a majority of the participants, our data suggest that it is possible to induce considerable metabolic stress by running merely 5 km, when striving for maximal speed.

## Background

To increase exercise frequency is often recommended in the non-pharmacological treatment of many components of the metabolic syndrome, for example to lower glucose levels in type 2 diabetes [1]. Several earlier studies have shown improved glycemic control with regular exercise interventions, as well as beneficial effects on insulin sensitivity, blood lipids and blood pressure following exercise in patients with impaired glucose tolerance or overt diabetes [2–4]. Exercise increases energy expenditure, and is thus expected to reduce body weight. Indeed, not only does the energy expenditure increase during the actual performance of exercise, but metabolic rate is increased for several hours after that the physical activity has ceased [5].

Cortisol is an established marker of the hypothalamic-pituitary-adrenal (HPA) axis the activity of which has previously been shown to increase acutely after high-intensity exercise [6]. Indeed, stress hormones including cortisol have hyperglycemic effects [7], which makes measurement of this hormone relevant in the context of exercise. Insulin induces glucose uptake in fat cells and in muscle cells by translocation of the specific GLUT4 glucose transporters to the plasma membrane [8], and it also reduces glucose production in the liver. Exercise can also induce insulin-independent effects that translocate GLUT4 transporters to the plasma membrane, hence allowing increased uptake of glucose [9]. One of the mediators for this is likely the enzyme AMP-kinase that can be stimulated by increased production of AMP from the ATP used by muscles [10, 11]. Consequently short-term strenuous physical activity could potentially induce activation of the HPA axis which leads to release of hyperglycemic hormones while it at the same time causes production of AMP that increases insulin-independent glucose uptake. There has been increased interest in the potentially unbeneficial effects with the metabolic stress following extreme long distance races such as marathons and triathlons [12]. To our knowledge the net effects of metabolic stress and exercise induced insulin-independent glucose uptake has not been investigated clinically following common running distances used in recreational exercise.

We performed a study of healthy subjects comparing one day of minimal exercise with a day that started with a running race of 5 km at individual maximal speed. The objectives were to study the impact from running 5 km on glucose, insulin, insulin sensitivity, cortisol, glucagon, Troponin T and metabolic rate.

## Methods

Subjects were recruited consecutively with local advertising at Linköping University in south-eastern Sweden. Subjects had to be healthy and to have experience of running for exercise purposes. Exclusion criteria were significant medical conditions, regular medication (except birth control pills), abnormal thyroid hormone levels and obesity. The study duration was from September 1 to November 30 2011. The participants were studied on two separate occasions with at least two days apart and arrived to the clinic after an overnight fast for either a 5 km outdoor running race at individual maximal speed, or to an initial corresponding period (45 minutes) of rest. The subjects were randomized to whether they started with the running-race or not by drawing ballots. They were instructed to run as fast as they could during the race. Baseline blood samples were collected and a measurement of metabolic rate by indirect techniques was done before the race, or prolonged rest. Venous- and capillary blood were also taken at approximately 08.30 h (i.e. before race on a race day), 09.15 h (after race on a race day, on a non-race day this was “prolonged rest”), 11.05 h, 12.45 h, 14.30 h and 17.35 h for analyses of hormones. Levels of cortisol in saliva was also measured at the time of going to bed, in the evening at home, by supplying the participants with dedicated sponges to collect the saliva.

The original method for analysis of salivary cortisol has been described earlier [13] and the present method had a total CV of 14% when analyzed by Salimetrics Enzyme Immunoassay (Salimetrics LLC, Carlsbad, CA, USA) applied on a Nexgen Four equipment (Adaltis, Milano, Italy). Plasma glucagon was measured by radio-immuno-assay with a polyclonal antibody at the Department of Clinical Chemistry, Karolinska University Hospital, Stockholm, Sweden, and the total CV was 18%. Serum cortisol was analyzed on a Siemens Centaur XP equipment (Siemens, Munich, Germany) and had a total CV of 7.8%. Serum insulin had a total CV of 3.4% and high sensitive TroponinT had a total CV of 4.0% and they were both analyzed by using a Cobas e 602 equipment, (Roche, Basel, Switzerland). Capillary plasma-glucose was measured with an Accu-Chek glucometer (Roche Diagnostics, Basel, Switzerland). Insulin sensitivity was calculated from the QUICKI index, 1/(log_10_insulin+log_10_glucose), according to Katz et al. [14].

A standard 12-lead electrocardiogram (ECG) was recorded after return from the race and compared with an ECG taken during the non-exercise day. The measurements of the metabolic rate were done using indirect calorimetry by a Deltatrac Metabolic Monitor (Datex-Ohmeda Corporation, Helsinki, Finland) according to the instructions of the manufacturer and as described earlier [15, 16]. The Deltatrac equipment was allowed to warm up and to stabilize before the beginning of each measurement session. Measurements of metabolic rate were performed at 08.00 h, 10.05 h, 14.00 h and 17.30 h. The measurements were performed with the provided Deltatrac hood while the subjects was resting on a bed [16]. The metabolic rate was measured for about 20 minutes on each occasion. A mean value of the five readings during the last five minutes was calculated, when readings had stabilized. A standardized breakfast consisting of a ham sandwich, yoghurt and orange juice was given to the participants after the race or the corresponding prolonged rest period. The breakfast contained a total of 560 kCal and was composed of 22 g protein, 15 g fat and 84 g of carbohydrates. Two of the participants were vegetarians and they were given the same meal, but without the ham on the sandwich. One participant had celiac disease and was given the same yoghurt and orange juice, but the sandwich was substituted with a non-gluten equivalent containing the same nutritional value. Lunch consisted of a pre-packaged, frozen meal of pepper steak, potatoes au gratin and red wine sauce containing a total of 468 kcal based on 19.5 g from protein, 27.3 g from fat and 37 g from carbohydrates. Vegetarians had the meat substituted with a vegetarian equivalent with the same nutritional value. Water was offered as a drink to all the meals.

### Ethics

The study was approved by the Regional Ethics Committee of Linköping and performed in accordance with the Declaration of Helsinki. Approval no: 2011/388-32. Written informed consent was obtained from all participating subjects. The complete study protocol was reported to clinicaltrials.gov NCT01274078.

### Statistics

Statistical estimates were calculated using IBM SPSS software (IBM Corporation, Somers, New York, USA). Differences between effects of the day with a race in the morning compared with non-exercise day were analyzed by linear mixed model after log transformation of data to ensure normal distribution. Data on race-day or rest-day were set as fixed parameters in these analyses and study subject was set as the random effect. Mean values and standard deviations are given in the text and error bars in the graphs are SEM. Ad Hoc comparisons of single time points of interest, within and between groups were done with Student’s paired 2-tailed t-test on log-transformed data. Statistical significance refers to 2-sided p ≤ 0.05. The sample size of the trial was calculated from the power to detect a potential increase of the metabolic rate by running. Based on the precision of recordings of metabolic rate done by us earlier [16], the study had >80% power to detect a 5% post-race increase of the metabolic rate.

## Results

**Fig 1:**
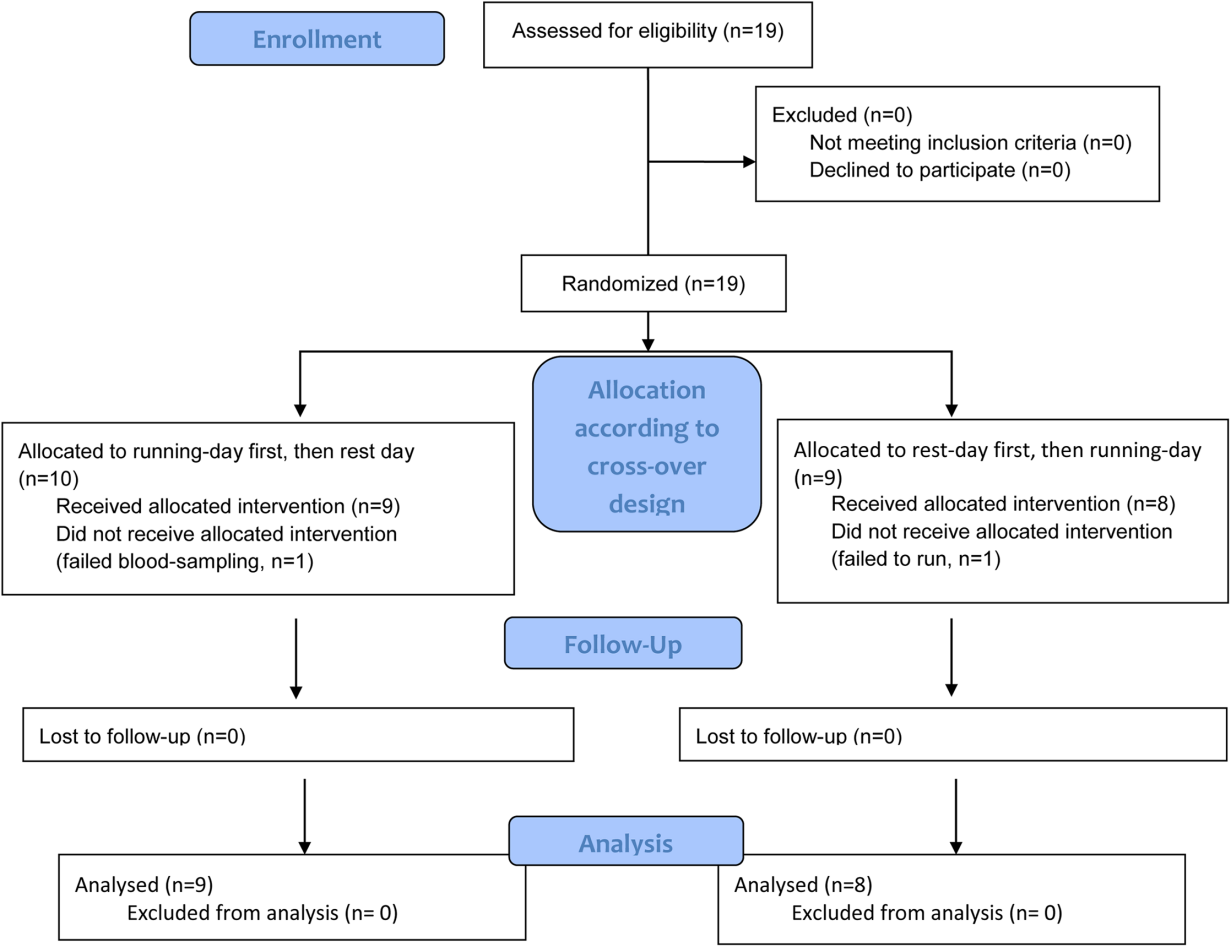
Consort flow diagram of the study.

Nineteen subjects were recruited for the study by local advertising. Five of the subjects had earlier participated in a pilot trial of the effects of regular exercise with or without supplementation with blueberries performed by us 6 months earlier [17]. In one of the subjects the venous cannula ceased to function soon after study start and numerous attempts of drawing blood failed. Since cortisol was a part of the study protocol, and since the patient was under stress and pain by failed attempts for venous access, further participation was judged to be meaningless. Another participant did not perform the 5 km race, and thus the corresponding rest-day was not used in the analysis of data. The remaining 17 subjects (5 women and 12 men) completed all tests on both the day of rest and the day that started with running 5 km after fasting for at least 10 hours (See Fig 1 flow diagram).

**Fig 2:**
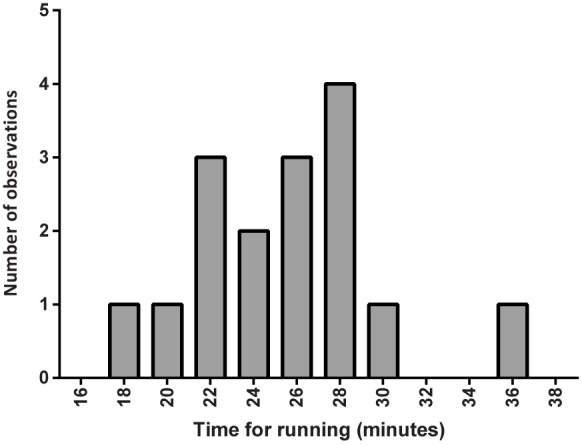
Histogram of the times it took for the participants to run 5 km at individual maximal speed. Data was missing in one subject for technical reasons.

The participants had no known current diseases judged to affect the trial, as specified by the inclusion criteria. One male participant was using sertraline, and one was treated with corticosteroid inhalations for asthma. All except three of the participants were university students and they were 25.7 ± 5.2 years of age (range 21–40 years) with a BMI of 22.5 ± 2.3 kg/m^2^ (range 19.6–28.4 kg/m^2^). Run times averaged 25.1 ± 4.3 minutes and as shown in Fig 2 there was a wide range in the time it took to finish the race. The heart rate at the ends of the races was on average 172 ± 20 beats/minute with a range from 126–200 beats/minute.

Effects of the race-day compared with non-exercise day on cortisol, insulin, glucose, glucagon, insulin sensitivity, metabolic rate, and Troponin T are shown in Table 1.

**Table 1:** Laboratory data during a day without exercise and a day which started with running 5 km at maximal speed (n = 17 participants). Data are mean values and (SD). Comparisons of data from the race-day with the day of rest was done with linear mixed model. Troponin T that was not detectable (limit of detection 5 ng/ml) was set as zero in calculations.

Variable	Rest orRace	Baseline	Before breakfast (i.e. after race on race-day)	Afterbreakfast	Beforelunch	After lunch	Evening	Plinear mixed model
Plasma glucose(mmol/l)	Rest	5.00 (0.68)	4.88 (0.59)	5.02 (1.6)	5.27 (0.52)	4.85 (0.55)	4.90 (0.62)	0.29
	Race	5.01 (0.37)	6.36 (1.3)	5.62 (0.73)	5.23 (0.69)	4.93 (0.60)	4.78 (0.54)	
Serum insulin(pmol/l)	Rest	36 (13)	33 (14)	164 (86)	91 (87)	78 (40)	34 (17)	< 0.0001
	Race	42 (21)	90 (54)	171 (59)	63 (34)	82 (39)	36 (25)	
QUICKI1/(log_10_insulin+log_10_glucose)	Rest	0.373 (0.037)	0.376 (0.028)	0.301 (0.027)	0.338 (0.040)	0.334 (0.023)	0386 (0.038)	< 0.0001
	Race	0.366 (0.030)	0.326 (0.037)	0.291 (0.016)	0.350 (0.039)	0.330 (0.021)	0.383 (0.038)	
Serum cortisol(nmol/l)	Rest	400 (157)	394 (187)	284 (129)	226 (92)	203 (92)	129 (59)	< 0.0001
	Race	408 (137)	644 (171)	369 (130)	254 (136)	211 (124)	166 (67)	
Salivary cortisol(nmol/l)	Rest	9.45 (5.4)	13.5 (12)	7.58 (4.6)	4.54 (1.8)	4.28 (2.3)	2.64 (0.88)	0.037
	Race	11.3 (4.9)	24.1 (10)	10.9 (5.4)	4.89 (1.9)	6.03 (6.4)	3.28 (1.1)	
Serum glucagon(pg/ml)	Rest	44.8 (6.5)	43.3 (8.9)	46.2 (6.1)	47.1 (7.6)	48.7 (8.9)	46.8 (4.5)	0.89
	Race	44.6 (6.5)	45.4 (7.5)	48.7 (7.7)	47.8 (6.1)	46.8 (8.4)	46.2 (7.4)	
Serum Troponin T (ng/l)								Not done
	Race	0 (0)	0 (0)	5.7 (6.1)	8.9 (8.8)	8.0 (9.0)	4.8 (6.2)	
Metabolic rate (kcal/24h)	Rest	1709 (236)	Not done	2044 (247)	Not done	1923 (290)	1800 (278)	0.53
	Race	1694 (266)	Not done	2245 (310)	Not done	1976 (286)	1811 (280)	

**Fig 3:**
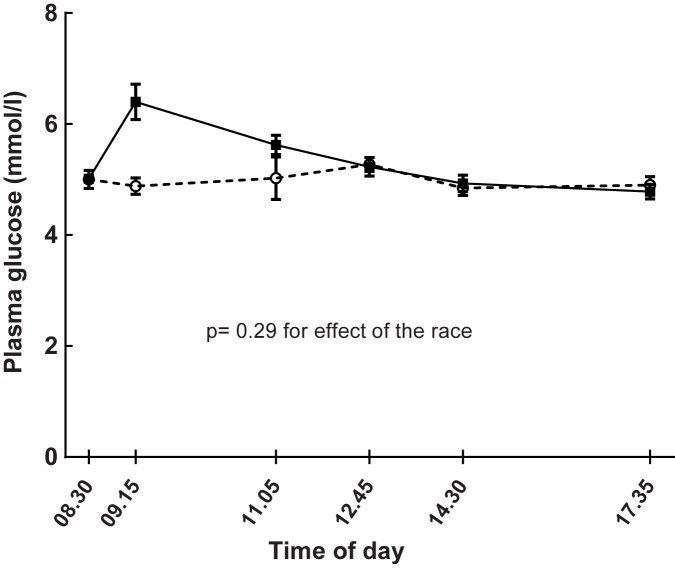
Effects of running (continuous line) 5 km on plasma glucose levels compared with a non-exercise day (dashed line) in 17 healthy subjects. The running took place after the baseline test on the day of the run. Data are means and SE. Linear mixed model for estimating the effects of the race: p = 0.29.

Plasma glucose levels were not generally higher on the race-day compared with a day of rest when analyzed by linear mixed model (Fig 3, Table 1, p = 0.29). However, plasma glucose increased from 5.01 ± 0.37 mmol/l at baseline to 6.36 ± 1.3 mmol/l (p <0.0001, range post-race from 4.9 to 9.9 mmol/l) by the running race (Table 1 and Fig 3). Correspondingly, the level of glucose after the race was higher when compared with the same time-point on the non-exercise day (6.36 ± 1.3 mmol/l vs 4.88 ± 0.59 mmol/l, p = 0.001).

**Fig 4:**
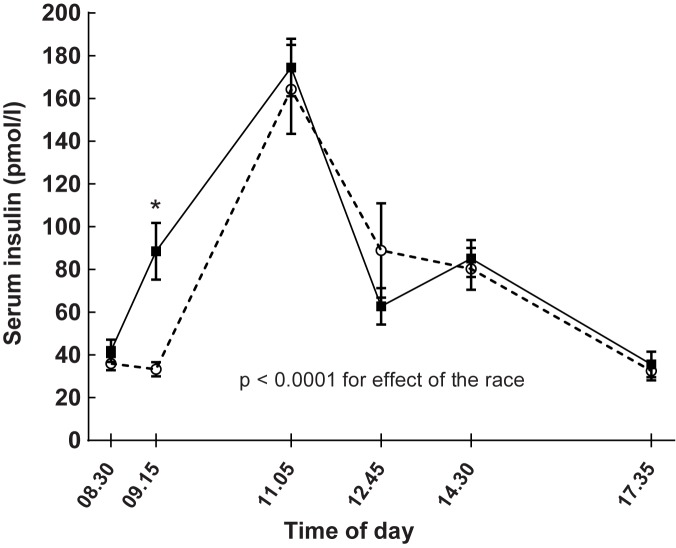
Effects of running 5 km (continuous line) on serum insulin levels compared with a non-exercise day (dashed line) in 17 healthy subjects studied on two separate occasions. Running took place after the baseline test on the day of the run. Data are means and bars reflect SEM. Linear mixed model for estimating the effects of the race: p < 0.0001. Serum insulin levels increased after the race on the race-day, * p < 0.0001.

Linear mixed model analysis showed that insulin levels were generally higher on race-days compared with days of rest (Table 1, p < 0.0001). As seen in Fig 4 and Table 1 serum insulin increased about two-fold following the races when compared with baseline levels (p < 0.0001). Correspondingly, post-race serum insulin was higher compared with the same time-point on the non-exercise day (Table 1, p < 0.0001).

**Fig 5:**
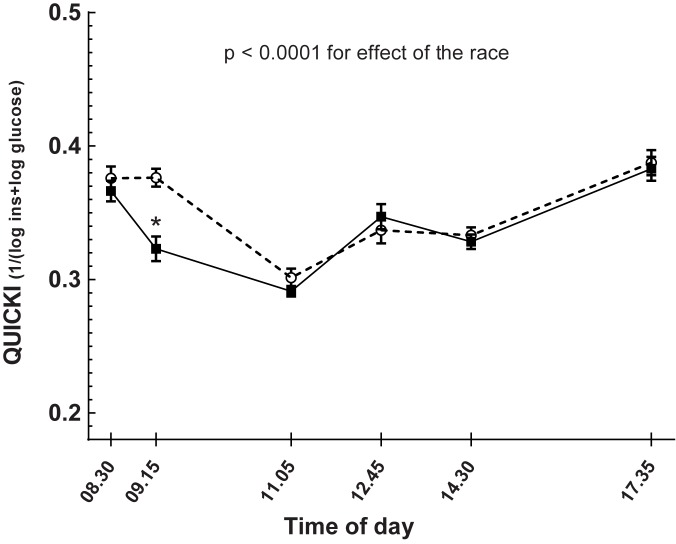
Effects of running 5 km (continuous line) on insulin sensitivity by QUICKI index 1/(log_10_insulin+log_10_glucose) compared with a non-exercise day (dashed line) in 17 healthy subjects studied on two separate occasions. Running took place after the baseline test on the day of the run. Data are means and standard errors. Linear mixed model for analyzing the effect of the race compared with the day of rest: p < 0.0001. *corresponds to p < 0.0001 when comparing QUICKI index before and after the race on the day of the race.

Insulin sensitivity by QUICKI showed reduced sensitivity for insulin during the days that started with exercise compared with the day of rest, as seen in Table 1 and Fig 5 (p < 0.0001). The QUICKI index fell after exercise compared with the baseline levels the same day (p < 0.0001) as seen in Fig 5.

**Fig 6:**
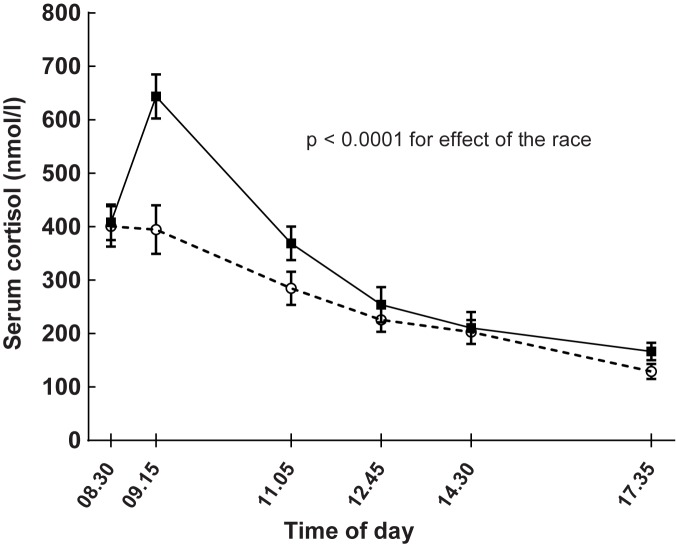
Effects of running 5 km (continuous line) on serum cortisol levels compared with a day without exercise (dashed line) in 17 healthy subjects. Running took place after the baseline test on the day of the run. Data are means and standard errors. Linear mixed model for estimating effects of the race: p < 0.0001.

Cortisol levels were higher on the race compared with the non-race day (linear mixed model, p-values for serum and for salivary cortisol p < 0.0001 and 0.037, respectively, Table 1 and Fig 6). Serum cortisol was higher during the race-day compared with the non-exercise day also at the last measurement in the evening (paired t-test, p = 0.013). Salivary cortisol that was measured before going to bed (not part of the Table 1) was higher on the race-day compared with the non-exercise day (rest-day: 1.58 ± 1.1 nmol/l, race-day: 2.22 ± 1.0 nmol/l, p = 0.015). However, salivary cortisol levels were similar at individual waking up times in the morning after the race (rest-day: 16.0 ± 5.5 nmol/l, race-day: 14.5 ± 5.3 nmol/l, p = 0.32). There were no correlations between delta salivary (r = 0.08, p = 0.8) or delta serum cortisol (r = 0.30, p = 0.12) and race-times.

The serum glucagon levels were unaffected by running (p = 0.89, Table 1).

**Fig 7:**
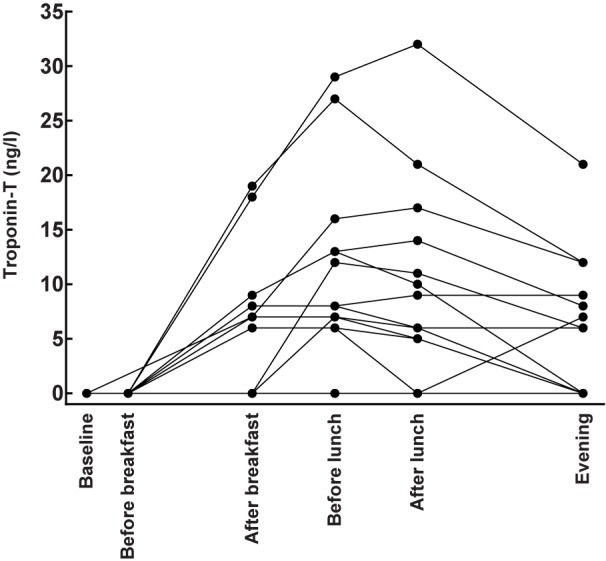
Effects of running 5 km on Troponin T in 17 healthy subjects. Individual consecutive changes in Troponin T are displayed by the aligned filled circles. Undetectable levels were set as zero in the graph.

Troponin T was not detectable in serum at baseline or immediately after return from the running (Table 1). The numerically highest mean values were seen before lunch when 12 out of 17 runners had detectable levels of Troponin T. The two highest recorded levels of Troponin T after the races were 32 ng/l and 27 ng/l respectively, which were abnormal levels according to the clinic laboratory reference level of 15 ng/l (Fig 7).

Troponin T levels were also measured 24 hours after the race and one participant had a detectable levels of the protein at this time point of 5 ng/l. ECGs that were recorded after the races showed no pathological changes.

**Fig 8:**
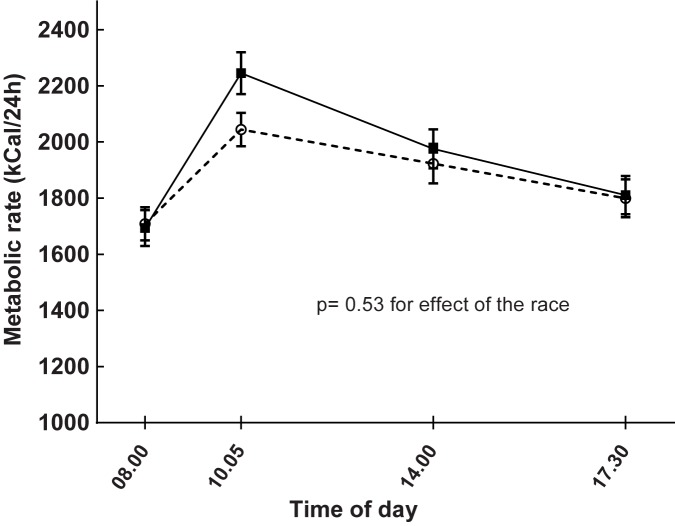
Effects of running 5 km (continuous line) on metabolic rate measured with indirect calorimetry compared with a day without exercise (dashed line) in 17 healthy subjects studied on these two separate occasions. Running took place after the baseline test on the day of the run. Data are means and standard errors. Linear mixed model for comparing the metabolic rate on the day that started with a race with the day without exercise: p = 0.53.

Metabolic rate did not generally differ on the day of the race as compared with the day of rest as seen in Fig 8 (linear mixed model: p = 0.53, Table 1). However, the metabolic rate was higher after the breakfast on the race-day compared with the same occasion on the day of rest (rest-day: 2044 ± 247 kcal/24h, race-day: 2245 ± 310 kcal/24h, p < 0.0001).

Serum cortisol levels post-race correlated positively with serum insulin at the same time point (r = 0.64, p = 0.005) and also with the increase of insulin levels by the race (delta serum insulin vs. post-race serum cortisol: r = 0.62, p = 0.001). The corresponding correlation with the increase in plasma glucose by the race was not statistically significant (delta plasma glucose vs. serum cortisol post-race: r = 0.41, p = 0.10).

## Discussion

We found that running 5 km at maximal individual pace temporarily increased glucose levels immediately after running. This increase was apparently not due to a lack of insulin response since serum insulin levels about doubled in parallel with the plasma glucose elevations. The running-races also increased the serum cortisol levels about 60% post-race compared with the non-exercise days (644 ± 171 nmol/l and 394 ± 187 nmol/l, respectively). Another suggestive sign of that increased activity in the HPA-axis reduced insulin sensitivity was the correlation between serum cortisol and serum insulin levels post-race. However, although cortisol reduces insulin sensitivity and increases de novo production of glucose, serum cortisol could potentially mostly have served as a marker of metabolic stress acting through epinephrine and/or norepinephrine release since these systems are often activated in parallel [18, 19]. It is also possible that release of growth hormone reduced insulin sensitivity and induced gluconeogenesis, as has been shown earlier during moderately intense exercise [20]. We acknowledge the short-coming of not being able to analyze all potentially important hormones that can affect insulin sensitivity and glucose production in this trial. We had a focus on cortisol since it serves as a good marker for mental and physical stress in relation to arteriosclerosis and insulin resistance [21–24]. By measuring salivary cortisol we were also able to analyze free cortisol late in the evening, in the home environment, and also when waking up in the morning after the races. We were thus able to demonstrate complete normalization of salivary cortisol in the morning after the races. We also acknowledge that the QUICKI index was developed for estimation of insulin sensitivity during fasting. However, lately it has been found to serve as a marker of insulin sensitivity also in the non-fasting state with reasonable precision [25]. The data based on the QUICKI equation in our trial were thus found to correspond with the global effects by the races to elevate insulin levels and the transient increase in glucose levels post-race.

Given that the races caused increased levels of both glucose and insulin post-race, the insulin-independent glucose uptake in muscle by exercise [10, 11] apparently did not fully compensate for the decreased insulin sensitivity by running. Also, since serum insulin levels immediately after the races were increased three-fold compared with the non-race day, cortisol did not strongly suppress insulin release in the beta-cells. This is otherwise a known mechanism by which cortisol can elevate plasma glucose [26, 27]. Our findings of reduced insulin sensitivity in healthy subjects following vigorous exercise were in line with the acute effects of a 10 sec race to prevent hypoglycemia following prolonged moderate exercise in patients with type 1 diabetes in a study by Bussau et al. [28]. Also Guelfi et al. have shown that intermittent short races reduce hypoglycemia following moderately strenuous exercise in patients with type 1 diabetes, while increasing norepinephrine and epinephrine levels [29]. However, these very short bouts of running did not increase cortisol levels [29], as was found in our study of running 5 km. In yet another related study Mc Mahon et al. exercised patients with type 1 diabetes for 45 minutes on bicycles, and found elevated levels of cortisol, growth hormone, and epinephrine [20]. However, insulin sensitivity increased both during and after this moderate-intensity bicycling, as judged by the need to increase glucose infusion rates [20]. Differences regarding insulin sensitivity compared with our study might be related to lower level of exertion, a longer duration of the exercise or that patients with type 1 diabetes have a different reaction pattern than the healthy participants of our trial [20].

We found that the metabolic rate measured with indirect calorimetry increased post-race, while there was no global such increase on race-days. Thus, the metabolic effect of the race to induce increased cortisol during the studied time-period was paralleled with a transient increase also in the metabolic rate. This was in line with earlier studies showing that lack of cortisol reduces the metabolic rate while iatrogenic hypercortisolemia increases energy expenditure in humans [30, 31].

Troponin T levels were detectable in a majority of participants after the races and levels peaked after about 4 hours. The dynamics of serum Troponin T in healthy participants following such a relatively short running distance as 5 km has to our knowledge not been assessed before. The data could be helpful when elevated serum Troponin T levels are evaluated clinically after exercise and differentiated from other possible causes such following myocarditis or trauma. Three participants in our study had Troponin T that exceeded the clinic reference level for myocardial injury. There is a discussion whether transient increases in serum Troponin T are caused by necrosis of myocardial cells or whether Troponin T is simply released by these cells upon stress [32]. However, much more strenuous exercise than in our trial has been linked with both myocardial scarring and arrhythmias [12, 33, 34]. In our study there were no changes in the ECGs post-race, and all but one of the participants had undetectable Troponin T levels the day after the race. We acknowledge the limitation of the study by not incorporating more exact functional myocardial analyses such as echocardiography. It was also a limitation that subjects without previous running experiences were not included, such future studies could indeed be of interest, given the results reported herein.

In conclusion we found that strenuous running for 5 km in healthy subjects can induce reductions in insulin sensitivity that are not fully compensated to keep plasma glucose levels constant despite substantially increased serum insulin levels. The decrease in insulin sensitivity following the races, as well as the elevated serum and salivary cortisol and Troponin T suggest that running 5 km at maximal speed might not have net effects that are advantageous in every respect from a cardiovascular perspective.

## Supporting information

S1 File Consort checklist. (DOC)

S2 File Translated research protocol. (DOC)

S3 File Research protocol (Swedish). (DOC)

S4 File Translated ethical application form. (DOCX)

S5 File Ethical application form (Swedish). (DOC)

S6 File Original data of the study, excel file. (XLSX)
