# Supplementary material for: A randomized cross-over study of the acute effects of running 5 km on glucose, insulin, metabolic rate, cortisol and Troponin T
Source: PLoS One. 2017 Jun 16;12(6):e0179401. doi: 10.1371/journal.pone.0179401 (PMC5473541; doi:10.1371/journal.pone.0179401)
Supplement: S2 File — (DOC) [file pone.0179401.s002.doc]

Linköping 16/3 2011

**For the Ethical Committee in Linköping
Application for a supplement concerning case / study 2010/121-31**

I have previously get the study "Effects of physical exercise on risk markers for cardiovascular disease," approved by the Ethics Board (2010 / 121-31). We found in this study, quite unexpected, that a majority of participants (75%) had elevations of cardiac-specific troponin T test after running workouts at 5km, indicating heart cells damage during the run. This finding was independent of whether they were physically fit or not, and was not affected by the training or "coach lying" in the study.

I wish now to call some of the participants again (about 12 people would be enough) to take samples more often than what we've done so far (in the first analysis, samples were taken only after about 12 hours) before and after the race to see when the maximum changes in blood samples occurs. It is clinically important to know this, to avoid wrongly diagnosis of pathological heart damage after running workouts among exercisers. We now wish to take samples at a total of eight times during a day, which also includes the study of metabolism in addition to previously inflammation, blood fats and other risk factors for heart disease. Thus, we wish to implement a new running race in addition to the four already made in the study, but with more frequent testing for, total sample volume would be <200ml. Since it takes some time, I would like to give 700 SEK in compensation for these additional examinations to those who stand up for this. We also planned to offer breakfast and lunch during this day.

The fee for this case (SEK 2000) is now set to your account.

In my judgment means an extra running workout in addition to the four already done no ethical problem but it can give us important information that we benefit of in clinical practice now when we found that the running of such a relatively short distance can cause noticeable changes in lab tests.

Fredrik Nystrom, Professor, Chief Physician
Study Manager, dept. KVM, IMH, HU
Email: fredrik.nystrom@lio.se
Phone: 0736 569 303

Appendix: previously approved application
