# Supplementary material for: A randomized cross-over study of the acute effects of running 5 km on glucose, insulin, metabolic rate, cortisol and Troponin T
Source: PLoS One. 2017 Jun 16;12(6):e0179401. doi: 10.1371/journal.pone.0179401 (PMC5473541; doi:10.1371/journal.pone.0179401)
Supplement: S3 File — (DOC) [file pone.0179401.s003.doc]

2026-08-29

**Till Etiska nämnden i Linköping**

Ansökan om en förändring av tidigare komplettering för ärende/studie 2010/121-31 och 2011/1234-32

Jag har tidigare fått godkänt att genomföra ett extra löpningslopp i den tidigare etiskt godkända ”träningsstudien” med diarienummer 2010/121-31. I denna träningsstudie jämfördes en period av 4 veckor löpträning med motsvarande tid utav minsta möjliga fysiska aktivitet. Studiens huvudsyfte uppnåddes på de 32 deltagarna under hösten 2010, det blev en reell skillnad i fasteinsulin och blodfetter i slutet av de båda perioderna. Det fynd som föranledde att vi ansökte om en ändring (Dnr 2011/1234-32) var emellertid att 75% av deltagarna fick detekterbart troponin-T efter det avslutande löpningsloppet som endast var 5 km långt. Troponin-T används kliniskt för att diagnostisera hjärtinfarkt, men i den ursprungliga studien analyserades detta prov bara vid en enstaka tidpunkt 12 timmar efter löpningen.

Vi har nu påbörjat undersökningen av Troponin-T efter ett löppass med mer tät provtagning i enlighet med den godkända förändringen av protokollet (Dnr 2011/1234-32). Huvudanalysen har gått bra på de första 5 som sprungit och 3 av dessa har visat en övergående kraftig stegring av troponin-T, som når över det idag aktuella referensvärdet på hjärtskada (> 15 ng/l) med maximum ca 4 timmar efter loppet. Fyndet är kliniskt mycket relevant då man således just 4 timmar efter träning (löpning) kan komma att överdiagnostisera hjärtskada om en potentiell hjärtpatient hamnar på akuten efter löpning.

Tyvärr så försvåras vår undersökning av att det blivit färre av de som ingått i den ursprungliga studien som haft tid att göra det extra loppet. Undersökningarna utförs ju på dagtid till skillnad från tidigare prover. Vi har nu endast 11 personer som kan tänka sig vara med i denna extraundersökning och det kommer göra vår analys statistiskt svag och därmed svårpublicerad. Men den tidigare godkända ändringsansökan var formulerad så att den bara gällde de som varit med i den tidigare 12 veckor långa studien. Jag ansöker nu om att få rekrytera ca 5 friska frivilliga (som givetvis inte får vara i beroendeställning till mig) för att mäta prover före och efter ett löpningslopp på morgonen, vilka inte varit med i den ursprungliga 12 veckor långa undersökningen. Det skulle i så fall ge oss ett totalt antal av ca 16 personer, vilket borde räcka för att kunna publicera detta kliniskt viktiga fynd att troponin-T ofta visar en övergående stegring med maximum ca 4 timmar efter ett 5 km löppass.

Avgiften för detta ärende (2000 kr) sätts idag in på ert konto.

Jag kan inte se att det är ett etiskt problem att låta andra friska frivilliga får genomföra detta löpningslopp med provtagning därefter, även om de inte varit med i den ursprungliga 12 veckor långa studien. Den enda risk med undersökningen är den som är relaterad till blodprovtagning, vilket innebär minimal risk. Jag bedömer alltså att riskerna är mycket små jämfört med möjligheten att kunna ställa en mer korrekt diagnos på hjärtskada om smärtor i bröstet uppstår i samband med fysisk aktivitet. Vi har redan friska frivilliga som är intresserade att delta, som hört talas om studien och anmält intresse, så någon annonsering eller motsvarande behövs inte för att i så fall kunna slutföra studien med tillräckligt stort antal deltagare.

Fredrik Nyström, professor, ÖL

Studieansvarig, avd. för KVM, IMH, HU

Email: [fredrik.nystrom@lio.se](mailto:fredrik.nystrom@lio.se)

Telefon: 0736 569303.

Bilaga: tidigare godkänd ändringsansökan
