# Supplementary material for: A randomized cross-over study of the acute effects of running 5 km on glucose, insulin, metabolic rate, cortisol and Troponin T
Source: PLoS One. 2017 Jun 16;12(6):e0179401. doi: 10.1371/journal.pone.0179401 (PMC5473541; doi:10.1371/journal.pone.0179401)
Supplement: S5 File — (DOC) [file pone.0179401.s005.doc]

# ANSÖKAN OM ETIKPRÖVNING

# Information till ansökan, *se bilaga och Vägledningar (*[*www.epn.se*](http://www.epn.se/)*)*

**Till Regionala etikprövningsnämnden i:** Linköping

Den regionala etikprövningsnämnd till vars upptagningsområde forskningshuvudmannen hör, se respektive nämnd *(*[*www.epn.se*](http://www.epn.se/)*)*

Avgift inbetald datum: Ja

Observera att en ansökan aldrig är komplett och därmed kan behandlas förrän blanketten är korrekt ifylld och avgiften är betald.

**Projekttitel:** Effekter av fysisk träning på riskmarkörer för hjärt- och kärlsjukdom

Ange en beskrivande titel på svenska för lekmän, utan sekretesskyddad information. Ange också i förekommande fall projektets identitet, projektets/forskningsplanens (protokollets eller prövningsplanens) nummer, version, datum osv.

Projektnummer/identitet:       Version nummer:

## **Uppgifter som fylls i av den regionala etikprövningsnämnden**

Ansökan komplett: Dnr:

Begäran om ytterligare information (i sak): Begärd information inkommen:

Beslutsdatum: Expeditionsdatum:

**Ansökan avser (gäller även vid begäran om rådgivande yttrande):**

Forskning där endast en forskningshuvudman deltar (5 000 kr)

Forskning där mer än en huvudman deltar (16 000 kr)

Forskning där mer än en forskningshuvudman deltar, men där samtliga

forskningspersoner eller forskningsobjekt har ett omedelbart

samband med endast en av forskningshuvudmännen (5 000 kr)

Endast behandling av personuppgifter (5 000 kr)

Forskning som gäller klinisk läkemedelsprövning (16 000 kr)

Ändring av tidigare godkänd ansökan enligt 4 § förordning (2003:615) om

etikprövning av forskning som avser människor (2 000 kr)

Om nämnden finner att forskningsprojektet inte faller inom etikprövninglagens tillämpningsområde

önskas ett rådgivande yttrande. [(Info: 4a och 4b §§ i förordning 2003:615)](http://www.epn.se/media/8604/2003_615_4ab.doc) [(Info: Bilaga till ansökan)](http://www.epn.se/media/8525/bilaga.doc)

Ja:  Nej:

**1. Information om forskningshuvudman m.m.**

**1:1 Forskningshuvudman** ([Info: p. 1:1 i Vägledning till ansökan](http://www.epn.se/media/8601/vta_p1_1.doc))

Ansökan om etikprövning av forskning ska göras av forskningshuvudmannen. *Med forskningshuvudman avses en statlig myndighet eller en fysisk eller juridisk person i vars verksamhet forskningen utförs.* Inom staten utförs forskning främst vid lärosätena, men även vid vissa andra myndigheter, som t.ex. Brottsförebyggande rådet och Socialstyrelsen. Kommuner och landsting kan vara forskningshuvudmän, liksom privaträttsliga juridiska personer.

Namn: Linköpings Universitet

Adress: HU, 581 85 Linköping

**1:2 Behörig företrädare för forskningshuvudmannen**

Behörig företrädare är t.ex. prefekt, enhetschef, verksamhetschef. Forskningshuvudmännen bestämmer själva, genom interna arbets- och delegationsordningar eller genom fullmakt, vem som är behörig att företräda forskningshuvudmannen. Kopia av sådan handling *ska* bifogas.

Namn: Toste Länne Tjänstetitel: Proprefekt, professor

Adress: Institutionen för Medicin och Hälsa, Hälsouniversitetet i Linköping, 581 85 Linköping

**1:3 Forskare som är huvudansvarig för genomförandet av projektet (kontaktperson)**([Info: p. 1:3 i Vägledning till ansökan](http://www.epn.se/media/8714/vta_p1_3.doc))

Namn: Fredrik Nyström Tjänstetitel: ÖL prof.

Adress: Institutionen för Medicin och Hälsa, HU, 581 85 Linköping

E-postadress: fredrik.nystrom@lio.se

Telefon: 013 227749

Mobiltelefon: 0736 569303

**1:4 Plats** ([Info: p. 1:4 i Vägledning till ansökan](http://www.epn.se/media/8717/vta_p1_4.doc))

Plats (er) där projektet ska genomföras, ange inrättning (ar), institution (er), klinik (er) etc.

Institutionen för Medicin och Hälsa, Hälsouniversitetet i Linköping, 581 85 Linköping

**1:5 Andra medverkande**

Övriga deltagande forskningshuvudmän samt forskare ansvariga för att lokalt genomföra projektet (kontaktpersoner) anges här eller i bilaga med namn och adresser (se p. 9 bilaga nr 1).

Elisabet Aardahl, läkare, disputerad på "stress", Klin. kem. lab., Linköpings Universitetssjukhus.

Torbjörn Lindström, endokrinolog/läkare, EM kliniken HU

Marta Vergara, ST läkare endokrinologi, EM kliniken, HU, doktorand med TL och FN som handledare.

Moa Palmqvist samt Sofia Nyberg, fördjupningsstudenter på läkarlinjen, kommer arbeta heltid med detta projekt under höstterminen 2010.

**1:6 Ansökan/anmälan till andra myndigheter**

**Vid läkemedelsprövning**

Ansökan om tillstånd av *Läkemedelsverket* – se Läkemedelsverkets hemsida ([www.mpa.se](http://www.mpa.se/) )

Ansökan inlämnad (datum)       Tillstånd erhållits

EudraCT nr:

**Vid viss genetisk forskning**

Om personuppgifter om genetiska anlag som har framkommit efter genetisk undersökning kommer att hanteras i studien ska detta anmälas till *Datainspektionen* enligt 10 § personuppgiftsförordningen (1998:1191) – se Datainspektionens hemsida [www.datainspektionen.se](http://www.datainspektionen.se/lagar-och-regler/personuppgiftslagen/forhandskontroll/)

Anmälan inlämnad (datum)       Kommer att inlämnas efter godkänd etikprövning

**Vid viss forskning som innefattar bestrålning av forskningspersoner** ([Info: p. 9 i Vägledning till ansökan](http://www.epn.se/media/8747/vta_p9.doc))

Ansökan, enligt 16 och 22 §§ Strålsäkerhetsmyndighetens föreskrifter (SSMFS 2008:35) om allmänna skyldigheter vid medicinsk och odontologisk verksamhet med joniserande strålning, till *Strålskyddskommitté* –
för vidare information kontakta aktuell lokal strålskyddskommitté.

Ansökan inlämnad (datum):       Ansökan tillstyrkt

### 2. Uppgifter om projektet

**2:1 Sammanfattande beskrivning av forskningsprojektet (programmet)**[Vägledning till forskningsplan/forskningsprotokoll (program)](http://www.epn.se/media/8929/vägledning till forskningsplan.pdf) ([Info: p. 9 i Vägledning till ansökan](http://www.epn.se/media/8747/vta_p9.doc))

Beskrivningen ska kunna förstås av nämndens samtliga ledamöter. Undvik därför terminologi som kräver specialkunskaper. Ange bakgrund och syfte för studien samt den/de vetenskapliga frågeställning (ar) som man söker svar på. Ange de viktigaste undersökningsvariablerna. Beskriv vilka kunskapsvinster projektet kan förväntas ge och betydelsen av dessa. Ange om det är en registerstudie, uppdragsforskning etc. För fackmän avsedd detaljerad information i forskningsplan/forskningsprotokoll (program) *ska* bifogas som bilaga (se p. 9 bilaga nr 2). En utförligare beskrivning av studiens genomförande *avsedd för lekmän* kan vid behov bifogas den för fackmän avsedda obligatoriska forskningsplanen.

Ordination av ökad fysisk aktivitet används ofta i vården i syfte att minska risker för hjärt- och kärlsjukdom. Trots det finns inga prospektiva randomiserade studier med undersökning av sjuklighet och död (så kallade "hard endpoints") som visat att sådana råd minskar sjuklighet. Vi har idag studier av hög kvalitet som talar för att man på populationsnivå och i kohorter kan prediktera risken för hjärt och kärlsjukdom med hjälp av flera markörer som kan mätas i serum/plasma. Utöver de klassiska riskfaktorerna som blodfetter och blodsocker utgör, finns det många som hävdar att låggradig inflammation, mätt såsom högsensitivt CRP (hs-CRP), är en stark riskmarkör för framtida hjärt- och kärlsjukdom. De som tränar regelbundet visar ofta lägre nivå av hs-CRP än otränade och fr.a. överviktiga individer. Samtidigt verkar det som att akut träning kan öka hs-CRP i viss mån, kanske genom att träningsvärk i sig associerar med en akut inflammation. Vi ämnar nu undersöka skillnader i olika riskmarkörer för hjärt- och kärlsjukdom, och hur dessa påverkas av om man är vältränad eller ej. Ca 30 friska och frivilliga personer kommer att genomgå en crossover-studie där man antingen under en månad tränar löpträning (5 pass/vecka, ca 5 km varje gång), sedan lever som vanligt under en månad och slutligen försöker avhålla sig ifrån fysisk aktivitet under en månad i så hög utsträckning som möjligt (soffliggande). Deltagarna lottas till vilken period de börjar med, träning eller soffliggande. Samtidigt lottas (1:1, randomiseras) hela gruppen till att antingen äta som vanligt eller att få tillägg av koncentrerad blåbärsdryck för att på detta naturliga vis konsumera antioxidanter (resveratrol fr.a.). Vi kommer att förse dessa deltagare med de frusna blåbären som kommer inköpas från COOP och vara av märket Findus (så att kvaliteten påverkas av årstiden). Prover på hjärt- och kärl sjukdomsriskmarkörer tas i början o slutet av varje period (träning eller soffliggande) före och efter den dag man utför ett löppass där man springer 5 km så fort man kan (försöksledarna är då med och kontrollerar att man tar ut sig maximalt). Deltagarnas hormonella reaktion (stegringen av kortisol, ett stresshormon) på mental stress kommer också att testas före och efter träning, där stressfaktorn utgörs av att utföra test såsom att utföra matematikuppgifter på tid (räkna baklänges t.ex., resultatet av hur snabbt man räknar/utför testet är inte det primära således, det är bara ett sätt att öka kortisolnivåerna) datorbaserade prov. Syftet med studien är alltså att undersöka om man påverkar riskmarkörer för hjärt och kärlsjukdom på individnivå då en period med soffliggande jämförs med en motsvarande period av relativt flitig träning på samma sätt. Vi undersöker också om mental stress hanteras olika om man är vältränad eller ej, samt om inflammation kan uppkomma av träning. Studien har betydelse för hur man skall tolka de ofta ganska svängande resultat man kan få utav prover som tas för att bedöma risk för kärlsjukdom samt ge ytterligare kunskap om vad inflammationsmarkören hs-CRP står för kliniskt.

Sammanfattningsvis är studien alltså prospektiv cross-over studie där man lottas till vilken av perioderna man börjar med, träning eller soffliggande. Alla 30 deltagarna lottas också till att få blåbärstillskott (naturliga antioxidanter) eller ej, dvs ca 15 personer kommer inta 1,5 dl frusna blåbär, lämpligen som en dryck, i samband med träningen, eller låta sina mat/dryckvanor vara oförändrade.

**2:2 Vilken/vilka vetenskaplig (a) frågeställning (ar) ligger till grund för projektets utformning?**

Om projektet kan karakteriseras som en hypotesprövning, ange den primära och eventuellt sekundära hypotesen. Hänvisning till mer detaljerad information för fackmän kan ske till bifogad forskningsplan enligt punkt 2:1

Påverkas riskmarkörer för hjärt och kärlsjukdom, såsom hs-CRP och faste-insulin av 4 veckors intensiv träning jämfört med motsvarande tid av låg fysisk aktivitet och påverkas detta i så fall av intag av antioxidanter i blåbär?

Reagerar men med lägre kortisolstegring vid mental stress om man är vältränad jämfört med om man varit fysiskt inaktiv?

**2:3 Redogör för resultat från relevanta djurförsök**

Om djurförsök inte utförts ange skälen till detta.

Ej relevant

**2:4 Redogör översiktligt för undersökningsprocedur, datainsamling och datas karaktär**

([Info: p. 2:4 i Vägledning till ansökan](http://www.epn.se/media/8720/vta_p2_4.doc))

Av beskrivningen ska framgå hur projektet planeras genomföras. Beskriv insamlade datas karaktär. Ange hur datas tillförlitlighet säkerställs (t.ex. kvalitetskontroll/monitorering). - Vid enkäter och intervjuer ska beskrivas tillvägagångssätt och t.ex. frågors innehåll och hur slutsatser dras. Enkäter och skattningsskalor *ska* bifogas (se p. 9 bilaga nr 5). - För medicinsk forskning ska anges t.ex. typer av ingrepp, mätmetoder, antal besök, tidsåtgång vid varje försök, doser och administrationssätt för eventuella läkemedel och/eller isotoper, blodprovsmängd (även ackumulerad mängd vid multipla försök). Ange även om och på vilket sätt undersökningsprocedur m.m. skiljer sig från klinisk rutin. Ange proceduren för att ge den eventuella behandling efter projektets slut, som kan erfordras. Ange procedur för insamling av biologiskt material. Redogör för datakällor och procedurer vid behandling av personuppgifter. För mer detaljerad information kan hänvisning ske till bilagd forskningsplan.

Studien innefattar enkätdata om hur man mår samt blodprover för att studera riskmarkörer och inflammation. Blodprover tas vid 8 tillfällen (se forskningsprogram). Vi samlar också in uppgifter om hur snabbt man klarar av att springa 5 km samt antropometriska data som kroppsvikt blodtryck, bukomfång mm. Följsamhet till träningen kommer monitoreras med träningsdagböcker och genom att försöksledarna helt enkelt är med och tränar. Under "soffliggperioden" kommer pedometrar, stegräknare, att användas.

**2:5 Redogör för om insamlat biologiskt material kommer att förvaras i en biobank** ([Info: p. 2:5 i Vägledning till ansökan](http://www.epn.se/media/8723/vta_p2_5.doc))

*Med biobank avses biologiskt material från en eller flera människor som samlas och bevaras tills vidare eller för en bestämd tid och vars ursprung kan härledas till den eller de människor från vilka materialet härrör.*Redogör för var och hur prover som ska sparas förvaras, kodningsprocedurer och villkor för utlämnande av prover. Ange huvudman för biobanken. Observera att i förekommande fall ska anmälan av biobank ske till Socialstyrelsen enligt lagen (2002:297) om biobanker i hälso- och sjukvården m.m.

Blod för senare analys av hormoner sparas i -80C frys som befinner sig på Farmakologen, US. Aktuella provrör med sådant material kommer att frysas och förses med koder för varje försöksperson. Fredrik Nyström har ansvaret för utlämning av frysta blodprover för analys. Data kommer att lagras och bearbetas avidentifierat.

**2:6 Redovisa tillgång till nödvändiga resurser under projektets genomförande**

Ange vem/vilka som har ansvaret (prefekt, verksamhetschef eller motsvarande) för forskningspersonernas säkerhet vid alla enheter/kliniker där forskningspersoner ska delta. Intyg från dessa ansvariga *ska* bifogas (se p. 9 bilaga nr 9). Av intyget ska framgå att erforderliga ekonomiska, strukturella och personella resurser finns tillgängliga för att garantera forskningspersonernas säkerhet.

Personella resurser på Universitetet finns för projektet, se intyg.

**2:7 Journalföring, registrering och hantering av data** ([Info: p. 2:7 i Vägledning till ansökan](http://www.epn.se/media/8750/vta_p2_7.doc))

Redogör för hur undersökningsprocedurer och eventuella ingrepp journalförs. Ange hur registrering och behandling av resultaten ska gå till. Om materialet ska kodas, ange proceduren, vem som förvarar kodlistor och vem eller vilka som har tillgång till dem, var och hur länge de förvaras samt om materialet kommer att anonymiseras eller förstöras. Ange om band- och videoinspelningar används. Redogör för vilken tillgänglighet datamaterialet har och hur det förvaras samt hur erforderligt sekretesskydd erhålls.

Kodlista för sammankoppling av avidentifierade data med faktiska personuppgifter/personnummer förs av huvudansvarig Fredrik Nyström. Inga ingrepp eller medicinska åtgärder utförs, och därmed sker ingen medicinsk journalföring i studien. Materialet kommer i original att sparas på Universitetssjukhuset. Avidentifierade datafiler kommer att hanteras inom universitetet (HU). Om avvikande prover som inger misstanke om sjukdom upptäcks hanteras detta på vanligt kliniskt vis och det hela handläggs på EM kliniken med sedvanlig journalföring och utredning utanför studien där Fredrik Nyström är ansvarig.

**2:8 Redogör för tidigare erfarenheter (egna och/eller andras) av den använda
proceduren, tekniken eller behandlingen**

Särskilt angeläget är att redovisning av risker för komplikationer görs tydliga och i förekommande fall med angivande av relevanta publikationer. Vid nya behandlingar av patienter, t.ex. med läkemedel, bör anges hur många patienter (med aktuell eller annan åkomma) som tidigare erhållit föreslagen behandling, läkemedelsdosering (eller annan dosering) samt hur långa behandlingsperioder som studerats.

Fredrik Nyström har utfört flera studier på friska frivilliga där motsvarande undersökningar utförts. Fysisk träning i form av löpning bör inte leda till risker för deltagarna som kommer rekryteras utifrån att de har erfarenhet av löpträning sedan förut. Blåbärsintag antas inte ge biverkningar.

**3. Uppgifter om forskningspersoner**

**3:1 Hur görs urvalet av forskningspersoner?** ([Info: p. 3:1 i Vägledning till ansökan](http://www.epn.se/media/8726/vta_p3_1.doc))

*Med forskningsperson avses en levande människa som forskningen avser.*Ange urvalskriterier (inklusion och exklusion). Redogör för på vilket sätt forskaren kommer i kontakt med/får kännedom om lämpliga forskningspersoner. Ange om rekrytering sker från egna/andras tidigare eller pågående studier. Om annonsering sker, *ska* annonsmaterialet insändas som bilaga (se p. 9 bilaga nr 3). Om t.ex. barn eller personer som tillfälligt eller permanent inte är kapabla att ge ett eget informerat samtycke ska ingå i projektet, ska detta särskilt motiveras. Om vissa grupper (t.ex. kvinnor, barn eller äldre) utesluts från deltagande i projektet ska detta särskilt motiveras.

Inklusionskriterier: Friska män och kvinnor, som är ca 20-35 år gamla och som har viss löpträningserfarenhet sedan tidigare men som inte är extremt vältränade vid studiestart.

Exklusionskriterier: allergi eller intolerans mot blåbär. Fysiska eller mentala problem som gör deltagandet i träning respektive soffliggande svårgenomförbart.

**3:2 Ange relationen mellan forskare/försöksledare och forskningspersonerna**

Behandlare (t.ex. läkare, psykolog, sjukgymnast) - forskningsperson (t.ex. patient, klient)

Kursgivare (lärare) - student

Arbetsgivare - anställd

Annan relation som kan tänkas medföra risk för påverkan. Beskriv: Forskare- frisk frivillig

3:3 Redogör för det statistiska underlaget för studiepopulationens (-ernas)/ undersökningsmaterialets (-ens) storlek [**(Info: p. 3:3 i Vägledning till ansökan)**](http://www.epn.se/media/8729/vta_p3_3.doc)

Redovisa statistisk styrka, så kallad ”power”- beräkning eller redovisa motsvarande överväganden som tydliggör studiens möjligheter att besvara frågeställningarna.

Studiestorleken bygger på en undersökning som publicerades i PNAS 2009 (Ristow et al. 2009 106: p 8665) där motsvarande träning under 4 veckor gav signifikant ökad effekt på insulinkänslighet i subgrupper på 10 personer, samt medförde en höjning av hormonet adiponectin som ses som en skyddsmarkör för kärlsjukdom. Detta bör göra det möjligt att kunna finna en motsvarande effekt av träningen och av den potentiella effekten av blåbärstillskott. Se också forskningsprotokoll

**3:4 Ange om forskningspersonerna kan komma att inkluderas i flera studier samtidigt
eller i annan/andra studie (-er) i nära anslutning till denna? I så fall vilken typ av forskning?** ([Info: p. 3:4 i Vägledning till ansökan](http://www.epn.se/media/8732/vta_p3_4.doc))

Nej det är inte avsett.

3:5 Vilket försäkringsskydd finns för de forskningspersoner som deltar i projektet?

Det åligger forskningshuvudmannen att kontrollera att befintliga försäkringar täcker eventuella skador som kan uppkomma.

Vi kan inte se att undersökningarna innnebär några reella risker, men patientförsäkringen gäller för problem orsakade vid provtagningen.

**3:6 Vilken ekonomisk ersättning eller andra förmåner utgår till de forskningspersoner
som deltar i projektet och när betalas ersättningen ut?** Utförligare beskrivning kan lämnas i bilaga. ([Info: p. 3:6 i Vägledning till ansökan](http://www.epn.se/media/8735/vta_p3_6.doc))

Ersättning för obehag och besvär. Belopp (före skatt):

Ersättning för förlorad arbetsinkomst  Ja  Nej

Reseersättning  Ja  Nej

Befrielse från kostnader för läkemedel  Ja  Nej

Befrielse från andra kostnader. Vilka?

Andra förmåner. Vilka? Ersättning för provtagning: 1000 kr totalt.

När betalas ersättningen ut? Efter genomförd studie.

Ingen ersättning betalas ut

### 4. Information och samtycke ([**Info: Forskningspersonsinformation**](http://www.epn.se/media/8598/forskningspersonsinformation.doc))

4:1 Proceduren för och innehållet i den *information* som lämnas då forskningspersoner tillfrågas om deltagande

Beskriv hur och när information ges och vad den innehåller. Ange vem som informerar. Normalt ska en kortfattad och lättförståelig skriftlig information ges. Denna skriftliga information *ska* bifogas ansökan (se p. 9 bilaga nr 4). Om ingen eller ofullständig information ges, måste skälen för detta noggrant anges.

Försökspersonerna informeras såväl muntligt som skriftligt (se bilaga) av den studierepresentant som träffar dem i rekryteringssyfte.

**4:2 Hur och från vem inhämtas *samtycke*?**

Beskriv proceduren; vem som frågar, när detta sker och hur samtycket dokumenteras. Utförlig redovisning är särskilt viktig då barn eller personer med nedsatt beslutskompetens ingår i studien, likaså vid studier av en grupp/grupper, t.ex. föreningar, organisationer, företag, kyrkosamfund, församlingar eller skolklasser.

Försökspersonerna informeras såväl muntligt som skriftligt av den studierepresentant som träffar dem i rekryteringssyfte. Dokumentation genom skriftligt samtycke, se bilaga.

### 5. Forskningsetiska överväganden

5:1 Redogör för de risker som deltagandet kan medföra samt möjliga komplikationer

Dessa kan vara t.ex. fysisk skada, smärta, obehag eller integritetsintrång som projektet innebär eller kan innebära. Ange vilka åtgärder som har vidtagits för att förebygga de risker som nämns ovan samt vilken beredskap som finns för att hantera sådana komplikationer. Ange vilka/de metoder som kommer att användas för att efterforska, registrera och rapportera oönskade händelser.

Deltagarna kan komma att drabbas av träningsvärk men vi anser inte detta vara ett problem av allvarlig art. Endast deltagare med tidigare erfarenhet av löpträning rekryteras för att undvika denna bieffekt. Riskerna med venprovtagning får anses mycket små i denna kohort.

5:2 Redogör för förutsebar nytta för de forskningspersoner som ingår i projektet

Erfarenhet av att deltaga i ett forskningsprojekt, kunskap om halten av olika kardiovaskulära riskfaktorer/blodprover.

5:3 Gör en egen värdering av förhållandet risk - nytta för de forskningspersoner som deltar

Vi bedömer att det vetenskapliga värdet av studien och intresset för den enskilda deltagaren är större än de mycket små risker försökspersonerna utsätts för.

**5:4 Identifiera och precisera om etiska problem t.ex risk - nytta i ett vidare perspektiv
kan uppstå inom eller genom projektet**

Här kan redovisas om exempelvis vissa grupper kan komma att utpekas/få hjälp som ett resultat av studien.

Om vi visar att antioxidanter i blåbär negativt påverkar nyttan av fysisk träning kan detta komma att påverka Vasaloppets upplägg.

### 6. Redovisning av resultaten

**6:1 Hur garanteras forskningshuvudmannen och medverkande forskare tillgång till data (anges vid t.ex. uppdragsforskning) och vem ansvarar för databearbetning och rapportskrivning?**([Info: p. 6:1 i Vägledning till ansökan](http://www.epn.se/media/8744/vta_p6_1.doc))

Fredrik Nyström ansvarar för datainsamling samt vetenskapliga rapporter i referee-granskad internationell tidskrift.

6:2 Hur kommer resultaten att göras offentligt tillgängliga? Kommer studien att insändas för publicering i tidskrift eller publiceras på annat sätt?

Ange i vilken form resultaten planeras offentliggöras samt tidsplan för detta.

Vetenskapliga rapporter i referee-granskade internationella tidskrifter.

6:3 På vilket sätt garanteras forskningspersonernas rätt till integritet när materialet offentliggörs/publiceras?

Redovisas resultat på statistisk gruppnivå? Beskriv procedurer eller metoder för avidentifiering/anonymisering.

Resultaten presenteras på gruppnivå. All presentation är helt avidentifierad.

7. Redovisning av ekonomiska förhållanden och beroendeförhållanden

Redovisning enligt punkterna 7:1-7:3 syftar till att tydliggöra alla direkta eller indirekta förhållanden, som kan tänkas påverka forskarens relation till forskningspersonerna (vid t.ex. informations-, samtyckes-, genomförandeprocedurer).

7:1 Vid uppdragsforskning

Ange uppdragsgivaren t.ex. ett företag (vid klinisk läkemedelsprövning eller prövning av andra nya produkter), en organisation eller en myndighet.

Namn: Ej aktuellt. Kontaktperson:

Adress:       Telefon/mobiltelefon:

Ange uppdragsgivarens relation till forskningshuvudmannen/medverkande forskare, t.ex.
anställningsförhållande

**7:2 Redovisa eventuella ekonomiska överenskommelser med uppdragsgivare eller**

**andra finansiärer (namn, belopp)**

Vid klinisk läkemedelsprövning bör hänvisning ske till ingånget avtal med sjukvårdshuvudmannen. Liknande överenskommelser kan förekomma vid annan uppdragsforskning och ska redovisas på samma sätt. Separata överenskommelser med den/de som ska genomföra forskningen ska redovisas. Belopp som kommer att erhållas för studien/ersättning till kliniken/genomföraren, vad ersättningen ska täcka och ev. belopp som erhålls per forskningsperson, ska också anges här (se p. 9 bilaga nr 12).

7:3 Redovisa forskningshuvudmannens, huvudansvarig forskares och medverkande forskares egna intressen

Här redovisas t.ex. aktieinnehav, anställning, konsultuppdrag i finansierande företag, eget företag som kan få (direkt eller indirekt) ekonomisk vinst av forskningen (se p. 9 bilaga nr 12).

Det finns inga kända sådana egenintressen i denna studie.

**8. Undertecknande**

Behörig företrädare för sökande forskningshuvudman enligt p. 1:2

Ort:       Datum:

Signatur: __________________________________________________________________

Namnförtydligande:

Tjänstetitel:

Undertecknad forskare som genomför projektet (kontaktperson) enligt p. 1:3 intygar härmed att forskningen kommer att genomföras i enlighet med ansökan

Ort:       Datum:

Signatur: _________________________________________________________________

Namnförtydligande:

Tjänstetitel:

**9. Förteckning över bilagor** ([Info: p. 9 i Vägledning till ansökan](http://www.epn.se/media/8747/vta_p9.doc))

Dokument som, i tillämpliga fall, ska bifogas *om inte motsvarande information finns i blanketten* har markerats med x. Markera de bilagor som skickas in med denna ansökan.

| **Insänd med ansökan** | **Bil nr** | Beskrivning | **Klinisk läkemedels-**  **prövning** | **Annan forskning** |
| --- | --- | --- | --- | --- |
|  | 1 | Deltagande forskningshuvudmän och medverkande forskare (kontaktpersoner) vid forskning där mer än en forskningshuvudman deltar. Info p. 1:5 | x | x |
|  | 2 | För fackmän avsedd forskningsplan, vid behov även för lekmän avsedd bilaga. Info p. 2:1 och i Vägledning till forskningsplan/forskningsprotokoll (program) | x | x |
|  | 3 | Annonsmaterial för rekrytering av forskningspersoner. Info p. 3:1 och i Vägledning till ansökan p. 3:1 | x | x |
|  | 4 | Skriftlig information till dem som tillfrågas. Info p. 4:1 och i Forskningspersonsinformation | x | x |
|  | 5 | Enkät, frågeformulär. Info p. 2:4 | x | x |
|  | 6 | Gemensam EU blankett (gäller fr.o.m. den 1 maj 2004), gäller även vid ändring. | x |  |
|  | 7 | Sammanfattning av protokollet på svenska | x |  |
|  | 8 | Prövarhandbok alt. bipacksedel/produktresumé/IB | x |  |
|  | 9 | Intyg från verksamhetschef/motsv. om resurser och om forskningspersonernas säkerhet. Info p. 2:6 | x | x |
|  | 10 | CV för forskare (samma som p. 1:3) med huvudansvar för genomförandet, redovisa forskarens (-arnas) kompetens av relevans för studien. Info i Vägledning till ansökan p. 1:3 | x | x |
|  | 11 | Beskrivning av ersättning till forskningspersoner. Info p. 3:6 och  i Vägledning till ansökan p. 3:6 | x | x |
|  | 12 | Överenskommelser med uppdragsgivare/finansiär om  t.ex. anställningsförhållanden, bidrag/ersättning till prövningsplats, sjukvårdshuvudman, forskningshuvudman eller forskare. Info p. 7:2 och p. 7:3 | x | x |

**Övriga bilagor som bifogas ansökan:**
